# Livestock-Associated Methicillin-Resistant *Staphylococcus aureus* ST9 in Pigs and Related Personnel in Taiwan

**DOI:** 10.1371/journal.pone.0088826

**Published:** 2014-02-13

**Authors:** Hsin-Wei Fang, Po-Hsing Chiang, Yhu-Chering Huang

**Affiliations:** 1 School of Medicine, Chang Gung University, Gueishan, Taoyuan, Taiwan; 2 Department of Pediatrics, Chang Gung Memorial Hospital at Linkou, Gueishan, Taoyuan, Taiwan; Rockefeller University, United States of America

## Abstract

**Background:**

A livestock-associated (LA) methicillin-resistant *Staphylococcus aureus* (MRSA) strain sequence type 398 (ST398) is found related to animals and humans in Europe and North America. To evaluate the nasal carriage of MRSA among pigs and related workers in Taiwan, we conducted this study.

**Methods:**

From June 25 to October 1 2012, a total of 641 and 100 nasal swabs were obtained from pigs and related workers, respectively, from 22 pig farms nationwide and 2 pig auction markets in Taiwan. All MRSA isolates were molecularly characterized.

**Results:**

Overall, the nasal carriage rate of MRSA was 14.4% for pigs and 13% for humans. The carriage rate for pigs younger than 3 months was significantly higher than those older than 3 months (25.4% vs. 5.8%, p<.001). Percentage of MRSA-positive pig farms was 59.1% (13/22). The carriage rate for pigs in large-scale herds (≥10000 pigs) was significantly higher than that in small-scale (34.3% vs. 7.0%, p<.001) and that in auction markets (3.8%). The carriage rate was 19.2% (10/52) for pig farm workers, and the rate in large-scale farms was significantly higher than that in small-scale (36.8% vs. 9.1%, p = .014). Except for 3 isolates from humans, the other 99 isolates belonged to sequence type (ST) 9. 83 of 89 isolates from pigs shared a common pulsotype, which was also shared by 6 isolates from humans.

**Conclusion:**

More than 10% of pigs and related workers in Taiwan carried LA-MRSA ST9 in nares and cross-species transmission of LA-MRSA was documented by molecular methods.

## Introduction

*Staphylococcus aureus* is a major cause of infections in both hospitals and communities, causing diseases ranging from mild skin infections to fulminant septicemia, and becomes increasingly resistant to methicillin. Methicillin-resistant *S. aureus* (RSA) is usually considered a hospital pathogen, but increasingly, it is acquired in the community [1]–[3]. According to the patients with or without health care-associated risk factors such as residence in long term care facility, recent hospitalization or surgery, indwelling catheter or hemodialysis, MRSA can be categorized into healthcare-associated (HA-MRSA) and community-associated (CA-MRSA) (2–4). CA-MRSA strains are genetically different from HA-MRSA, such as limited antibiotic resistance, possessing Panton-Valentine leukocidin genes, carrying type IV or V staphylococcal cassette chromosome etc. [2]–[4]. However, various MRSA clones have spread between community and hospitals, particularly CA-MRSA transmitted in hospital settings, making the distinctions between CA-MRSA and HA-MRSA blurred [4].

In addition to being a human pathogen, *S. aureus* causes an array of infections in economically important livestock animals, particularly in pigs [5]–[7]. A specific MRSA sequence type 398 (ST398) is found related to various animals and humans across European countries and North America (8–12). ST398 appears to be largely pig- or veal-calf-associated, but the clone is characterized by the capacity to colonize multiple host species, including cows, sheep and poultry, may facilitates the colonization in animal workers and those whom contact with and can cause severe infections in humans. Several cases of ST398 infection in humans have been reported in European countries, Hong Kong and China [10], [13]–[17].

Although MRSA prevailed in Taiwan, including healthcare settings and community, for decades, the reports regarding LA-MRSA have been scanty [18]–[20]. Hence, we conducted a study to evaluate MRSA colonization among pigs and related workers according to the locations and pig cultivation scales of pig farms and the age of pigs surveyed. Molecular characteristics of the MRSA isolates were also analyzed.

## Materials and Methods

### Ethic statement

The study was approved by the institutional review board of Chang Gung Memorial Hospital and a written informed consent was obtained from each participant. The owners of the pigs gave permission for their animals to be used in this study.

### Sample collection

According to the statistic data of the Council of Agriculture, Executive Yuan, Taiwan (November 2011), there were 9619 pig farms raising 6,247,946 pigs in Taiwan. Of these pig farms, there were 1797 farms raising at least 1000 pigs, 176 farms raising at least 5000 pigs, and 53 farms raising at least 10000 pigs. A cut-off point of 10,000 pigs was defined as a border between a large-scale pig farm and a small-scale pig farm referring to the document published by Council of Agriculture, Executive Yuan in November 2011 (http://www.coa.gov.tw/show_index.php).

In this study, 22 pig farms and 2 pig auction markets in Taiwan were selected for sampling during June 25– October 1 2012. These 22 pig farms were located in 6 counties with different cultivate scales ranging from 1,000∼30,000 pigs. From each farm, a mean of 25 samples were collected, including grower pig (older than 1 month), sow and piglet (younger than 1 month). Two auction markets situated at two different counties were selected and included in this study. In each auction market, 4–5 pigs were randomly selected from each railing to represent each pig farm. Related animal workers including pig farm workers, auction market employers and regular visitors (suppliers, other pig farm workers) were invited to participate in this study.

Nasal specimens were collected with a sterile cotton-tip swab inserting into both anterior nares of each pig and human, and immediately transferred into the amie media of Copan Venturi Transystem® (108C.USE, Copan Diagnostics, Italy). The ill pigs assessed by veterinarian of each pig farms were excluded from selection. With labels indicating the geographic origin (county) and the category of specimen, the sample were then sent to laboratory of Chang Gung Memorial Hospital at Linkou under room temperature within 48 hours.

### Isolation of MRSA

Each specimen was incubated at 37°C overnight with 5% sheep blood agar plate (BD Diagnostics, Sparks, MD). Initially, each specimen was inoculated on both sheep blood agar palte and chromogen plate. Since the yielding rate of MRSA was similar for both methods, we used sheep blood agar plate only subsequently. Based on the patterns of beta-hemolysis and the macroscopic appearance, the suspected colonies of *S. aureus* were further incubated with 5% sheep blood agar plate at 37°C overnight. Repeated subcultivation of specimen was performed as needed if the incubations were mixed with multiple unrecognized colonies. After incubation and subcultivation, coagulase test were conducted by using rabbit plasma to ensure the identification of *S. aureus*. Cefoxitin test was then used to distinguish the MRSA from methicillin-susceptible *S. aureus* (MSSA) based on the recommendation of Clinical and Laboratory Standards Institute (CLSI) document M100–S17 [21]. All MRSA isolates were finally stored at −80°C.

### Susceptibility test

The susceptibility test was performed on Mueller–Hinton agar with disk-diffusion method following the protocol of CLSI. After incubating at 37°C for 24 hours, the minimal inhibition concentration (MIC) of antibiotics, including vancomycin, teicoplanin, linezolid, doxycycline, penicillin, ciprofloxacin, erythromycin, fusidic acid, clindamycin, and sulfamethoxazole-trimethoprim were then interpreted by CLSI document M100–S17 [21]. Quality control was achieved by *S. aureus* ATCC 29213 simultaneously.

### Molecular analysis

All the MRSA isolates were molecularly characterized. Pulsed-field gel electrophoresis (PFGE) with *Sma*I digestion was used to fingerprint MRSA isolates according to the procedure described previously [22], [23]. The genotypes were designated in alphabetical order, as in the previous studies [22], [23]); any new genotype, if identified, was designated consecutively. PFGE patterns with fewer than 4-band differences from an existing genotype were defined as subtypes of that genotype [22], [23]. The Staphylococcal cassette chromosome *mec* (SCC*mec*) typing was determined by two multiplex PCR strategy described previously [24], [25] and our previous studies [22], [23]. Control strains for SCC*mec* types I, II, III and IVa, kindly provided by Dr Keiichi Hiramatsu, were as follows: type I, NCTC10442; type II, N315; type III, 85/2082; and type IVa, JCSC4744. SCC*mec* typing for type V_T_ was determined by a particular primer described elsewhere [22], [23] and the strain TSGH-17 was used as control. The presence of Panton-Valentine leucocidin (PVL) genes were determined by a PCR strategy described previously [26]. Multilocus sequence typing (MLST) [27] and *spa* gene typing [28] was examined for some strains of representative PFGE patterns.

### Questionnaire

Each participant was asked to fill a questionnaire for risk factors identification, including demographic and clinical information. The demographic data contained age, gender, occupation, seniority, smoking habit, contact of second-hand smoke, animal-related occupation of family members, medical-related occupation of households and visit times of slaughterhouse during one year. The clinical data included diabetes mellitus, hypertension, current usage of tubes (nasogastric tube, tracheostomy tube, drainage tube, Foley catheter, port-A and dialysis tube) of family members or himself, recent hospitalization or surgery history within one year of family members or himself and antibiotics use within one month. Each pig farm manager was also demanded to fill a questionnaire to assay local circumstances factors, including cultivation scales, cultivation density, operate year, frequency of sterilization and concurrent animal cultivation.

### Statistical analysis

SPSS 18.0(SPSS Inc., Chicago, IL, USA) statistical software was utilized for statistical analysis. A *p* value <0.05 is considered statistically significant. The MRSA prevalence from demographic data, clinical data, information of each pig farm, antimicrobial susceptibility test for each antibiotic, pig age, pig categories (piglet, sow, grower pig) and human categories (pig farm worker, auction market employer, regular visitor) were examined by chi-square test. If there existed risk factors with a *p* value <0.05, multivariate logistic regression model would be further performed.

## Results

### Prevalence of MRSA

A total of 641 and 100 nasal swabs were collected from live pigs and related animal workers, respectively. Totally, 89 (14.4%) MRSA isolates were identified from 641 pigs, which included 536 pigs in pig farms (157 piglets, 158 sows, and 221 grower pigs) and 105 grower pigs in auction markets. No MSSA was identified from pigs. For pigs in auction market, only two (1.9%) MRSA isolates were identified from one of the two markets sampled. The carriage rate of MRSA for piglets (29/157, 18.5%), and grower pigs (50/326, 15.3%) was significantly higher than that for sow (10/158, 6.3%) (p<.01, odds ratio (OR) 3.35 [95% confidence interval (CI) 1.57–7.15] and 2.68 [95% CI 1.32–5.44], respectively). By age, 10 grower pigs were excluded owing to insufficient information of definite age. For pigs younger than 3 months of age, the carriage rate of MRSA was 25.4% (67/264), a rate significantly higher than that for those older than 3 months (22/377, 5.8%) (*p*<0.001, OR 5.49 [95% CI 3.29–9.16]) (Table 1).

**Table 1:** Methicillin-resistant *Staphylococcus aureus* prevalence among different groups of pigs and humans in Taiwan.

Group	% (positive/total)	*p* value
Pigs	14.4 (89/641)	
Younger than 3 months	25.4 (67/264)	<0.001
Younger than 1 month	18.5 (29/157)	
1 to 3 months	35.5 (38/107)	
Older than 3 months*	5.8 (22/377)	
3 to 5 months	7.7 (7/91)	
Older than 5 months	3.4 (4/118)	
Sows	6.3 (10/158)	
Human	13 (13/100)	
Pig farm worker	19.2 (10/52)	0.03
Auction market employer	0.0 (0/32)	
Regular visitor	18.8 (3/16)	

* Samples coming from 10 grower pigs in the subdivision were excluded due to insufficient information of definite age.

Percentage of MRSA-positive pig farms was 59.1% (13/22). The carriage rate for pigs in large-scale herds (raising ≥10000 pigs) was significantly higher than that in small-scale ones (34.3% vs. 7.0%, p<.001, OR 6.88 [95% CI 4.13–11.45]) and that in auction markets (1.9%). The detailed carriage rates of MRSA among pigs stratified by cultivated scales are shown in Table 2. Moderate positive correlation was found between the approximate cultivation scale and prevalence of MRSA (*p*<0.020, *r* = 0.500).

**Table 2:** Comparison of nasal methicillin-resistant *Staphylococcus aureus* carriage among 536 pigs and 52 related workers in 22 pig farms in Taiwan by different cultivation scales.

Cultivation scale (pig)	Pig farms (positive/collected/nationwide)	Pig* (positive/total)				Worker# (positive/total)
		Total	Piglet	Sow	Grower pig	
Above 20,000	2/2/14	21/66	6/10	0/10	15/46	3/6
10,000∼19,999	4/5/39	41/115	14/41	4/34	23/40	4/13
5,000∼9,999	2/4/70	12/126	5/29	1/30	6/67	3/8
3,000∼4,999	1/4/158	1/90	1/31	0/39	0/20	0/22
2,000∼2,999	3/4/255	11/57	3/26	4/22	4/9	0/2
1,000∼1,999	1/3/1208	1/82	0/20	1/23	0/39	0/1
Total	13/22/1744	87/536	29/157	10/158	48/221	10/52

*The carriage rate for pigs in large-scale herds (raising ≥10000 pigs) was significantly higher than that in small-scale ones (34.3% vs. 7.0%, p<.001, OR: 6.88 [95% CI 4.13–11.45]).

#The carriage rate for humans in large-scale herds was significantly higher than that in small-scale ones (36.8% vs. 9.1%, *p* = 0.014, OR: 5.83 [95%CI 1.29–26.38]).

Among 273 pig-related workers invited for participation, 100 workers, including 52 pig farm workers, 32 auction market employers, and 16 regular visitors, agreed to donate the nasal specimens, with a partition rate of 36.6%. 13 MRSA isolates (13.0%) and 6 MSSA isolates were detected among the 100 nasal specimens. The carriage rate was 19.2% (10/52) for pig farm workers, 0% (0/32) for auction market employers and 18.8% (3/16) for regular visitors. The carriage rate for human in large-scale farms was also significantly higher than that in small-scale ones (36.8% vs. 9.1%, p = .014, OR 5.83 [95%CI 1.29–26.38]). Female gender is the only risk factor (*p* = 0.027) observed after analyzing the demographic and clinical information [Table 3]. The odds ratio of MRSA colonization of female to male is 4.26 (95% CI 1.18–15.39).

**Table 3:** MRSA prevalence among related workers in aspect of demographic and clinical data.

Characteristics	No. (%) of subjects*		OR	95% CI	*p* value
	MRSA (n = 13)	Non-MRSA (n = 87)			
Age					
Younger than 30 yrs	1 (7.7)	14 (16.1)	0.25	0.02–3.25	0.265
30–45 yrs	4 (30.8)	28 (32.2)	0.50	0.08–3.31	0.466
45–60 yrs	4 (30.8)	31 (35.6)	0.45	0.07–2.98	0.400
Older than 60 yrs	2 (15.4)	7 (8.0)	Ref.	–	–
Gender					
Female	5 (38.5)	11 (12.6)	4.26	1.18–15.39	0.027
Male	8 (61.5)	75 (86.2)	Ref.	–	–
Occupation					
Pig farm worker	10 (72.9)	42 (48.3)	1.03	0.25–4.32	0.002
Auction market employer	0 (0)	32 (36.8)	–	–	–
Regular visitor	3 (23.1)	13 (14.9)	Ref.	–	–
Seniority					
Less than 5 years	2 (15.4)	26 (29.9)	1.15	0.10–13.82	0.910
5–9 year	4 (30.8)	10 (11.5)	6.00	0.58–61.84	0.132
10–14 year	1 (7.7)	15 (17.2)	Ref.	–	–
At least 15 years	6 (46.2)	35 (40.2)	2.57	0.28–23.25	0.401
Diabetes					
Yes	0 (0)	4 (4.6)	Ref.		
No	13 (100)	82 (94.3)	1.05	1.00–1.10	0.427
Hypertension					
Yes	1 (7.7)	12 (13.8)	Ref.	–	–
No	12 (92.3)	74 (85.1)	1.95	0.23–16.36	0.533
Smoking habit					
Yes	3 (23.1)	29 (33.3)	Ref.	–	–
No	10 (76.9)	57 (65.5)	1.70	0.43–6.64	0.444
Contact of second-hand smoke					
Yes	6 (46.2)	37 (42.5)	1.14	0.35–3.66	0.832
No	7 (53.8)	49 (56.3)	Ref.	–	–
Animal-related occupation of family members					
Yes	2 (15.4)	23 (26.4)	0.50	0.10–2.42	0.380
No	11 (84.6)	63 (72.4)	Ref.	–	–
Medical-related occupation of households					
Yes	2 (15.4)	2 (2.3)	7.64	0.98–59.81	0.026
No	11 (84.6)	84 (96.6)	Ref.	–	–
Visit times of slaughterhouse within one year					
Less than 10 times	9 (69.2)	30 (34.5)	1.50	0.41–5.54	0.541
10–19 times	4 (30.8)	20 (23.0)	Ref.	–	–
20–29 times	0 (0)	1 (1.1)	–	–	–
At least 30 times	0 (0)	35 (40.2)	–	–	–
Current usage of tubes of households or himself					
Yes	0 (0)	0 (0)	Ref.	–	–
No	13 (100)	86 (100)	0.16	0.00–8.20	0.36
Hospitalization or surgery history within one year of households or himself					
Yes	1 (7.7)	8 (9.2)	0.81	0.09–7.09	0.851
No	12 (92.3)	78 (89.7)	Ref.	–	–
Antibiotics use within one month					
Yes	0 (0)	11 (12.6)	0.24	0.01–4.38	0.338
No	13 (100)	75 (86.2)	Ref.	–	–

OR: odds ratio; CI: confidence interval.

*Full demographic and clinical data were not available for all participants.

Female is the only risk factor found among these demographic and clinical data, the odd ratio of which is 4.26 (95% CI1.18–15.39) with *p* value 0.027.

### Antimicrobial resistance

All 102 MRSA isolates from 89 pigs and 13 humans were sensitive to vancomycin, teicoplanin, linezolid and fusidic acid, and resistance to penicillin. The resistant rates of other antimicrobial agents to MRSA isolates from humans were different from those from pigs and the rates were as followings: doxycycline (30.8%, 85.4%), ciprofloxacin (76.9%, 100%), sulfamethoxazole-trimethoprim (23.1%, 15.7%), erythromycin (84.6%, 87.6%), clindamycin (92.3%, 100%). Statistical significance was noted for doxycycline (*p*<0.001), ciprofloxacin (*p*<0.001) and clindamycin (*p* = 0.009) between both groups.

### Molecular characteristics

Of the 102 isolates, a total of six pulsotypes with 41 subtypes were identified by PFGE. Only two types were identified for 89 isolates from pigs, with one major type for 83 isolates. All 6 pusotypes were identified for the isolates from humans.

For SCC*mec* types, only 6 isolates from humans were determined as type IV. The other 96 isolates carried class C *mec* gene complex but no *ccr*C gene complex was detected and thus could not be determined as either SCC*mec* types, which was similar to those reported from Taiwan (18,19). Only one isolate from humans carried PVL genes. 39 isolates and 16 isolates, respectively, with representative pulsotypes were selected for *spa* typing and MLST. Three sequence types (ST) and 5 *spa* types were identified. Except for 3 isolates from humans, the other isolates belonged to ST 9. Most MRSA isolates from pigs were *spa* type t899. Two isolates characterized as ST 59–SCC*mec* IV were common human community-associated MRSA in Taiwan and one isolate characterized as ST 30–SCC*mec* IV was relatively uncommon human strain in Taiwan. The detailed characteristics of MRSA isolates from human are shown in Table 4 and the distribution of molecular characteristics of MRSA isolates from both humans and pigs is shown in Table 5.

**Table 4:** Molecular characteristics of 102 MRSA isolates, categorized by pulsed-field gel electrophoresis (PFGE) patterns.

PFGE pattern	No.	SCC*mec*	PVL genes	MLST (No.)	*Spa* typing (No.)	Sample origin (No.)
AG	1	IV	–	30 (1)	t019 (1)	Human (1)
BX	89	UT	–	9 (10)	t899 (28), t1939 (2), t4358 (1)	Human (6), pig (83)
BY	3	IV	–	9 (1)	t899 (2)	Human (3)
BZ	7	UT	–	9 (2)	t899 (1), t1939 (2)	Human (1), pig (6)
C	1	IV	–	59 (1)	t437 (1)	Human (1)
D	1	IV	+	59 (1)	t3527 (1)	Human (1)

UT: untypeable, SCC: staphylococcal chromosome cassette, PVL: Panton-Valentine leukocidin, MLST: multilocus sequence type.

**Table 5:** Molecular characteristics and antimicrobial resistance of 13 MRSA isolates from humans.

PFGE	Group	SCC*mec*	PVL genes	MLST	*Spa* typing	Antimicrobial resistance
AG2	Visitor	IV	–	30	t109	P
BX	Worker	UT	–			P, CIP, E, CC
BX1	Worker	UT	–			D, P, CIP, E, CC, SXT
BX3	Worker	UT	–			P, CIP, E, CC
BX8	Worker	UT	–	9		P, CIP, E, CC
BX14	Worker	UT	–	9	t899	D, P, CIP, E, CC
BX18	Visitor	UT	–		t899	P, CIP, CC
BY	Worker	IV	–	9	t899	D, P, CIP, E, CC, SXT
BY1	Worker	IV	–			P, CIP, E, CC, SXT
BY2	Worker	IV	–		t899	D, P, CIP, E, CC
BZ3	Worker	UT	–	9	t1939	P, CIP, E, CC
C3	Worker	IV	–	59	t437	P, E, CC
D62	Visitor	IV	+	59	t3527	P, E, CC

D: doxycycline, P: penicillin; CIP: ciprofloxacin; E: erythromycin; CC: clindamycin; SXT: sulfamethoxazole-trimethoprim, UT: untypeable, SCC: staphylococcal chromosome cassette, PVL: Panton-Valentine leukocidin, MLST: multilocus sequence type.

## Discussion

Results from the present study showed that the prevalence of MRSA among pigs in Taiwan was 14.4%, which was lower than that for Western countries but higher than that for Asian countries such as China (58/509, 11.4%) [29], Korea (21/657, 3.2%) [30], Malaysia (1.4%) [31], and Japan (0.9%) [32], except that for Hong Kong (16%–21.3%) [33], [34]. The rate of MRSA carriage among pigs reported from Taiwan recently was 4% of 126 pigs from three different randomly chosen pig farms and one slaughterhouse in 2011 [18] and 42.5% of 299 pigs from 150 farms from 11 counties in western Taiwan at the second largest swine auction market with pigs ready for slaughter in 2009–2010 [19], respectively. As shown in the present study, nasal MRSA carriage rate among pigs was affected by the sampling location (from pig farms or auction market), the cultivate scales of pig farms and the age of pigs sampled. The time point and the methods of samplings may also affect the carriage rate.

All of MRSA isolates from pigs in the present study were ST9 based on MLST. MRSA ST9 was first detected and reported in Italy in 2010 [35]. However, rather than ST9, it is ST398, which was first reported from pigs [7], and prevailed among pigs in European countries, USA, Canada, as well as Korea [8]–[15], [30]. In Asia, CC9 (ST9 and single-locus variants), but not ST398, has also been reported predominantly in swine-associated environments in some countries such as China, Hong Kong, Japan, Thailand and Malaysia [29], [31]–[34], [36]. In Taiwan, reported recently [18]–[20], most MRSA isolates from pigs were characterized as sequence type ST9, non-SCC*mec* types I to VII, PVL-negative, *spa* type t899, and resistant to erythromycin, clindamycin, ciprofloxacin and gentamicin. All these characteristics were shared by the isolates from pigs in the present study, which suggest that this LA-MRSA clone had spread among pigs in Taiwan for years. In Japan, the main strain was found to be ST221 [32], while ST398/t034 and ST541/t034 were predominant in Korea [30]. ST9 differs from ST398 not only in geographic distribution but also in its enterotoxin profile and resistance to a broader range of antimicrobials.

In the present study, the resistant rates of antimicrobial agents to MRSA isolates from pigs were significantly higher than those from humans. Current quinolones used in pig farming in Taiwan include nalidixic acid, oxolinic acid, danofloxacin, enrofloxacin and orbifloxacin. Though fluoroquinolones are not used directly in pig farming, MRSA isolated from pigs might require ciprofloxacin resistance through other quinolone regimens. Among the tetracycline antibiotics, chlortetracycline, oxytetracycline, tetracycline and doxycycline are commonly used. Although clindamycin is not allowed to be used in pig farming, lincomycin is widely used. The usage of these antibiotics in pig farming may affect the antibiotic resistant patterns of MRSA isolates from these pigs.

Though the LA-MRSA isolates in most Asian countries shared a common sequence type, namely ST9, the molecular characterizations for these LA-MRSA isolates were not same at all. The LA-MRSA isolates dominant in China [29] were characterized as *spa* type t899–SCC*mec* III, t899 also for isolates in Hong Kong [33], [34], and t899–untypable SCC*mec* for Taiwan [18]–[20]). While, the isolates dominant in Malaysia were *spa* type t4358–SCC*mec* V [31] and t337–SCC*mec* IX for isolates in Thailand [36].

The rate of nasal MRSA colonization among pig farmers and/or slaughterhouse workers in the present study was 13%, a rate lower than that in European countries (20–45%), but higher than that in Asian countries, such as 5.5% (5/90) in Malaysia [31], and 1.7% (2/120) in China [29]. In the present study, workers in large-scale pig farms had a higher carriage than those in small-scale ones. Previous studies indicated that higher density increased the risk of MRSA colonization [35], [37], [38], which could not be demonstrated in the present study since this information was not obtained from the farmers. Female was the only risk factor of carrying MRSA in the current study, which was opposite to others [39], [40]. Low participation rate (36.6%) of the pigs-related workers might affect the survey results. It is also intriguing that no MRSA strain was identified from 32 auction market employers.

Reported from northern Taiwan recently, the rate of nasal MRSA carriage was 3.8% for 296 patients receiving hemodialysis [41], 3.8% for 502 adult patients visiting emergency room [22] and 3.8% for 3098 adults for health examination [42]. More than 80% of these isolates were characterized as ST 59–SCC*mec* IV or V_T_. In the present study, 3% of 100 pig-related workers carried MRSA of ST59 and ST30, which was in consistent with previous reports from Taiwan [43]. While, nasal MRSA colonization was identified for 10 additional participants and all MRSA isolates from them belonged to ST9. Moreover, PFGE pattern of these human MRSA isolates was same and was indistinguishable with that of MRSA isolates from pigs. Apparently, the LA-MRSA jumping from pigs to humans was documented, providing evidence that human contact with pigs would increase the risk of LA-MRSA colonization. In addition, one of the regular visitors was found to harbor MRSA of ST9, suggesting the probability of ST9 spread via human contact instead of animal contact, a scenario demonstrated for MRSA ST398transmission in Europe [40], [44].

Human infections of LA-MRSA ST398 have been reported in Europe since first identified from pigs [7], [14], [15], [45]–[47]. While, despite the prevalence of MRSA ST9 among pigs in Asia, infections caused by this clone in animals and humans were not addressed in the literature. However, Wan et al [22] found that of the isolates collected from the Taiwan Surveillance of Antimicrobial Resistance (TSAR), a biennial national surveillance program, five human clinical isolates of ST9–t899–PVL-negative were identified and were isolated in 1998, 2004, 2006 (2 isolates), and 2010; and 3 were from outpatients and 2 were from inpatients. A highly homogeneous virulence genotype and genomic profiles were identified among the ST9 MRSA isolates of human and swine origin, suggesting a recent common ancestor and implying cross-species adaptation. As LA-MRSA has the potential to colonize humans and its virulence may change over time, ongoing surveillance is needed to detect changes in epidemiology.

There are several limitations in the present study. First, the density of pigs in the farms was not obtained from the farm owners. Second, this survey was conducted at only one time point, so we could not understand whether the colonization of LA-MRSA among the workers was transient or persistent. Third, for those with LA-MRSA colonization, we did not further obtain nasal samplings from their household members to figure out whether the transmission of LA-MRSA occurred in the households. Fourth, the participation rate of pig-related workers was too low (around one-third) to better understand the perspective of LA-MRSA colonization among this population. Finally, in addition to the cultivation scale, the pig farms were chosen arbitrarily if the owners agreed to participate in the study after contact. The interpretation of the results from this study should be judged by this potential selection bias.

## Conclusions

A substantial proportion (>10%) of pigs in Taiwan harbored LA-MRSA, namely ST9–spa type t899, and the carriage was related to cultivation scales and age of pigs. A substantial proportion of pig-related workers also harbored LA-MRSA, which documented cross-species transmission of LA-MRSA in Taiwan. To better understand the epidemiology and transmission of LA-MRSA, further studies are needed.
